# Disentangling MRI-derived blood–brain barrier leakage into vascular permeability and surface area

**DOI:** 10.1177/0271678X261485415

**Published:** 2026-08-26

**Authors:** Damon Verstappen, Joost JA de Jong, Paulien HM Voorter, Maud van Dinther, Robert J van Oostenbrugge, Julie Staals, Jacobus FA Jansen, Walter H Backes

**Affiliations:** 1 Department of Radiology & Nuclear Medicine, Maastricht University Medical Centre+, Maastricht, The Netherlands; 2 Mental Health and Neuroscience Research Institute (MHeNs), Maastricht University, Maastricht, The Netherlands; 3 Department of Neurology, Maastricht University Medical Centre+, Maastricht, The Netherlands; 4 Cardiovascular Research Institute Maastricht (CARIM), Maastricht University, Maastricht, The Netherlands; 5 Department of Electrical Engineering, Eindhoven University of Technology, Eindhoven, The Netherlands

**Keywords:** Blood–brain barrier, cerebral small vessel disease, dynamic contrast-enhanced MRI, permeability, surface area

## Abstract

In neurovascular and neurodegenerative diseases, blood–brain barrier (BBB) disruption can be subtle and diffuse, and MRI-derived measures are confounded by microvascular architecture. Therefore, we aimed to disentangle the MRI-derived blood–brain barrier leakage rate (*K_i_*) into intrinsic vascular permeability (*P*) and surface area (*S*). In this cross-sectional study, 45 patients with cSVD and 26 elderly controls underwent dynamic contrast-enhanced (DCE) MRI to determine *K_i_* and vessel architecture imaging (VAI) to estimate *S*. These measures were used to calculate *P*. Compared to normal-appearing white matter (NAWM), *K_i_* was higher in WMH (β = 0.297, *p* < 0.001) and deep gray matter (DGM; β = 0.210, *p* = 0.007). *P* was higher in WMH (β = 0.410, *p* < 0.001) and similar between NAWM and DGM. *S* was smaller in WMH (β = −0.467, *p* < 0.001) and larger in DGM (β = 0.540, *p* < 0.001). *K_i_* (β = 0.289, *p* = 0.007) and *P* (β = 0.249, *p* = 0.021) were only higher in patients than controls in concentric shells surrounding WMH. *S* was smaller in WMH of patients than controls (β = −0.085, *p* = 0.049). With age, *K_i_* (β = 0.260, *p* = 0.008) and *P* increased (β = 0.273, *p* < 0.001), while *S* decreased (β = −0.079, *p* = 0.025). Disentangling *P* and *S* from the BBB leakage rate provides two biologically more specific and interpretable measures, which have counteracting effects on the BBB leakage in aging and disease.

## Introduction

**Figure 1.**
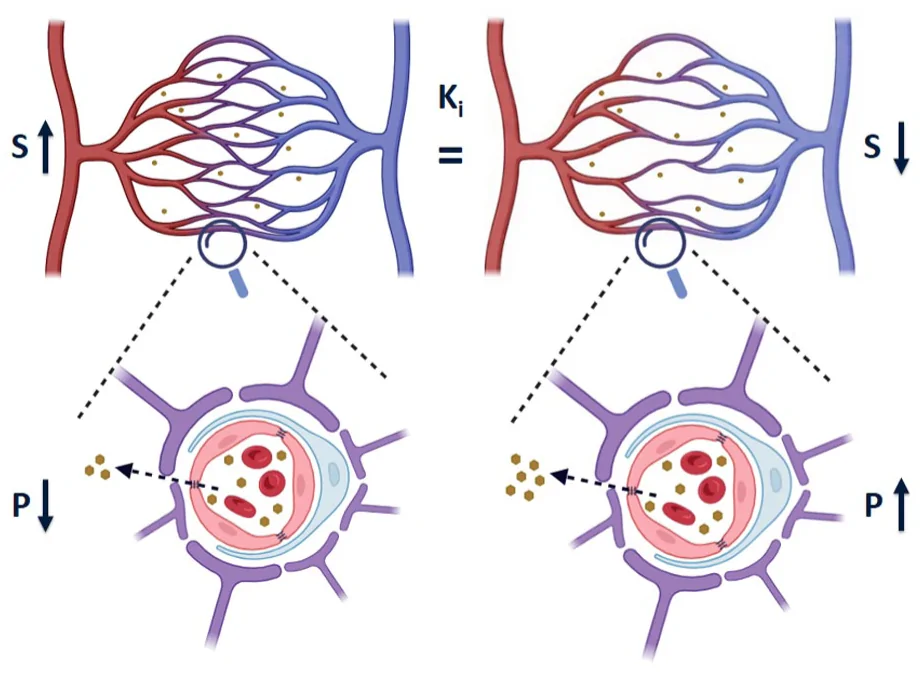
The counteracting effect of vascular surface area (*S*) and intrinsic vascular permeability (*P*) on the blood–brain barrier leakage rate (*K_i_*). Conceptually, a similar leakage rate can be obtained by the combination of a higher *S* and lower *P* as a lower *S* and higher *P*.

Contrast-enhancement in brain tissue is generally considered as evidence of blood–brain barrier (BBB) breakdown.^1^ The degree of BBB disruption is commonly determined by dynamic contrast-enhanced (DCE) MRI and pharmacokinetic modeling, yielding the leakage rate (*K_i_*) as a key quantitative BBB integrity (bio)marker.^2^ However, for neurovascular and neurodegenerative brain diseases and also aging, this leakage may not only be subtle and hard to determine,^3^ but also confounded by the underlying microvascular density.^2^ In particular, microvascular rarefaction as co-morbid brain pathology or aging phenomenon may obscure BBB permeability increases due to counterbalancing effects of increased intrinsic permeability and decreased microvascular surface area on *K_i_* (Figure 1).^3,4^ In line with this, higher *K_i_* in gray matter (GM) than in white matter (WM) is commonly ascribed to higher microvascular density in GM.^5,6^ Consequently, regional *K_i_* differences should not be unambiguously attributed to BBB breakdown, but also changes in microvascular density.

Currently, no explicit measurement techniques are available to measure either the intrinsic permeability or the surface area of cerebral microvessels.^2^ However, with vessel architecture imaging (VAI), it has recently become possible to measure the microvascular density and average vessel radius, which has been histologically validated in a number of studies.^7–9^ VAI is an advanced MRI perfusion technique, which exploits the differential sensitivity of spin-echo (SE) and gradient-echo (GE) sequences to various vessel sizes.^7^ This work uses knowledge of vessel sizes in combination with cerebral blood volume (CBV) to estimate the microvascular surface area (*S*) and to derive the intrinsic vascular permeability (*P*).

By separating vascular permeability from surface area, this study provides novel images of microvascular properties in patients with cerebral small vessel disease (cSVD) and elderly, for which a substantial range in microvascular pathology can be expected. We set out to establish the variation of *P* and *S* across major brain tissue types and evaluate the association of cSVD and age with these microvascular measures. In particular, we evaluated the heterogeneity of intrinsic permeability around characteristic white matter hyperintensities (WMH) presumed to be caused by variations in the underlying small vessel damage.^10^

## Methods

### Study population

Data were acquired from the miCRovascular rarefaction in vascUlar Cognitive Impairment and heArt faiLure (CRUCIAL-VCI) study (trial registration: ISRCTN22301128).^11^ In this prospective, cross-sectional study, patients with cSVD, who presented with vascular cognitive impairment (VCI) or a lacunar infarction were recruited from the memory or stroke clinic of the Maastricht University Medical Center+, The Netherlands. Recruitment took place between December 2020 and October 2024.

The definition of VCI due to cSVD was: subjective complaints of cognitive functioning, objective cognitive impairment in at least one cognitive domain in neuropsychological assessment or a Montreal Cognitive Assessment (MoCA) score <26, and imaging evidence of cSVD^12^ (extensive leukoaraiosis on CT, or moderate to severe WMH on MRI (Fazekas score ⩾2), or mild WMH (Fazekas score = 1) in combination with lacunes or microbleeds).^13^ Inclusion criteria for the lacunar stroke due to cSVD group were: a classical lacunar stroke syndrome with a compatible lesion on CT or MRI, and additional imaging evidence of cSVD (mild to severe WMH and/or microbleeds). Patients were scanned >3 months after the stroke to minimize acute post-stroke effects. Finally, we included a group of age-and-sex-matched controls from the neurology outpatient clinic where they presented with minor compression mononeuropathy or lumboischialgia. These controls did not undergo MRI prior to inclusion. Therefore, covert signs of cSVD were not ruled out, but the controls had no overt cognitive impairment or overt cerebrovascular disease.

Exclusion criteria included a Clinical Dementia Rating score >1,^14^ other neurological or psychiatric conditions affecting the brain and general contraindications for MRI or to tolerate gadolinium-based contrast agents. This study was approved by the Medical Ethics committee of the Maastricht University Medical Centre (approval number: NL72696.068.20). All participants provided written informed consent in accordance with the Declaration of Helsinki.

### MRI acquisition

All images were acquired using a 3 T MRI system (Philips Ingenia CX; Philips Healthcare, Best, The Netherlands) equipped with a 32-element head coil. The imaging protocol included two separate dynamic contrast-enhanced pulse sequences, one for DCE–MRI and the other for VAI.

The DCE–MRI protocol consisted of one 1-mm resolution pre-contrast T1 map and two post-contrast T1 maps spaced >20 min apart, to improve sensitivity to subtle leakage.^15^ These maps were acquired using a double inversion time (TI) gradient echo (GE) sequence (TR = 8.3 ms, TE = 3.8 ms, TI_1_ = 650 ms, TI_2_ = 2100 ms, FA = 6°, matrix size = 240 × 240, voxel size = 1 mm^3^). Hemodynamics were determined using a fast saturation recovery 3D DCE scan (TR = 5.3 ms, TE = 1.6 ms, saturation prepulse time delay (TD) = 120 ms, FA = 30°, matrix size = 128 × 128, voxel size = 2 × 2 × 6 mm, number of dynamics = 70, dynamic scan interval = 1.46 s) during contrast agent administration.^10^

For VAI, a rapid transverse single-shot combined GE SE echo-planar-imaging (EPI) sequence (Gyrotools LLC, Zurich, Switzerland; TR = 1634 ms, TE_GE_ = 25 ms, TE_SE_ = 78 ms, FA = 90°, matrix size = 76 × 61, voxel size = 3.0 × 3.3 × 5 mm, slice gap = 2 mm, number of dynamics = 100, dynamic scan interval = 1.6 s) was used.^16^ Before the dynamic series, one GE and one SE volume with the opposite phase-encoding direction were acquired to enable geometric EPI distortion correction.^17^

**Figure 2.**
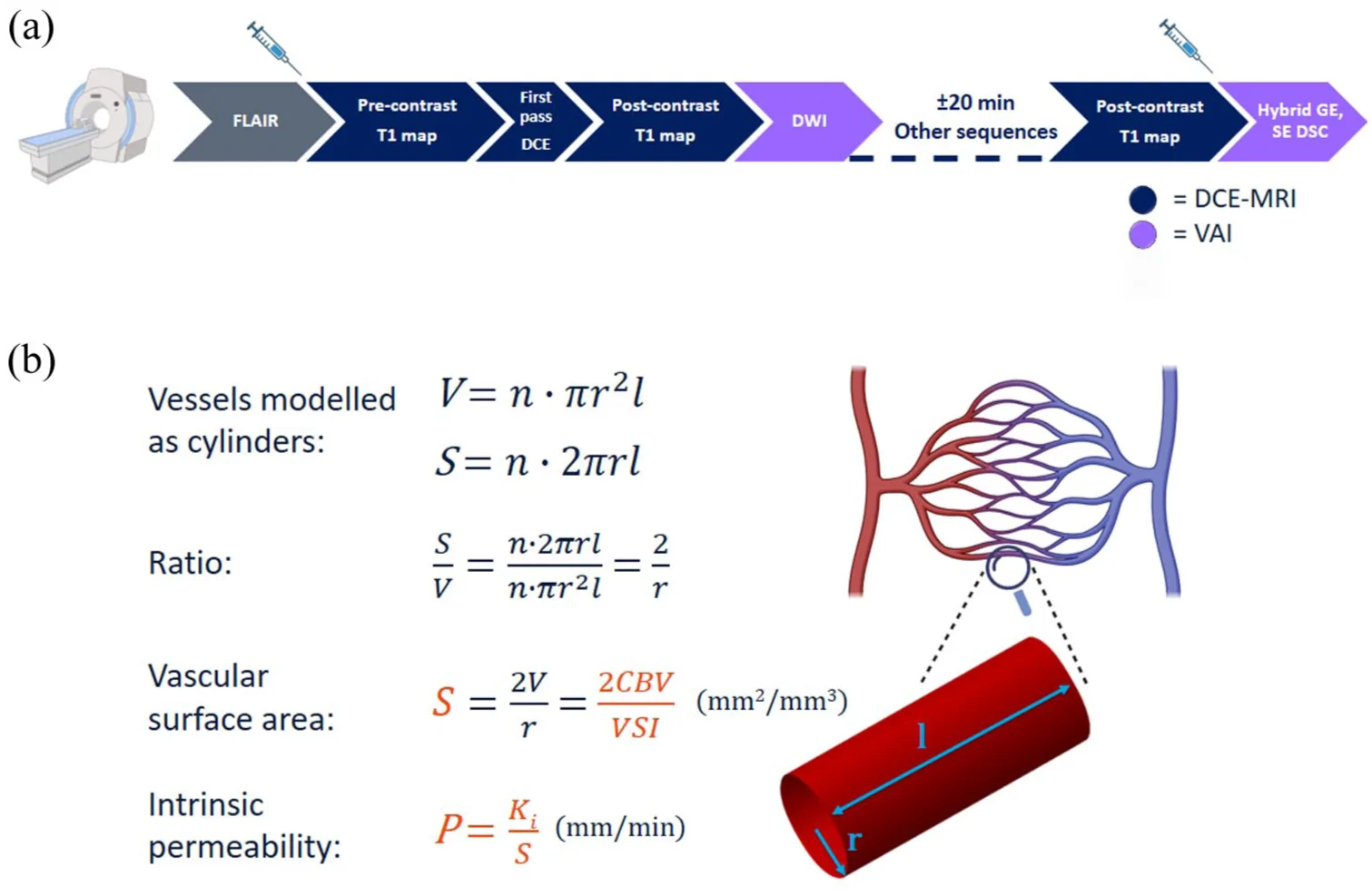
(a) MRI protocol timeline. The syringes denote the moments of contrast injection and (b) derivation of vascular surface area (*S*) and intrinsic vascular permeability (*P*). With a fixed CBV, a lower VSI leads to higher *S*. Note that the number of (*n*) microvessels is modeled with a homogeneous distribution of vessel sizes. CBV: cerebral blood volume; DCE: dynamic contrast enhanced MRI; DWI: diffusion weighted imaging; GE: gradient echo; SE: spin echo; DSC: dynamic susceptibility contrast; VAI: vessel architecture imaging; *V*: volume; *r*: radius; *l*: length; *K_i_*: blood–brain barrier leakage rate; VSI: vessel size index.

Both dynamic pulse sequences used a contrast agent dose of 0.1 mmol gadobutrol (Gadavist^®^) per kg body weight injected at 3 mL/s. The first dose, used for DCE–MRI, was administered approximately 30 min before the VAI scan and acted as a preload to reduce contrast agent-based T1 effects.^18^ No additional post-processing for T1-leakage correction was applied. In addition, diffusion-weighted imaging was performed to calculate the tissue’s diffusion coefficient and a T2-weighted fluid-attenuated inversion recovery (FLAIR) image was acquired for visualization of WMH (pulse sequence parameters: Supplemental Table S1 and protocol overview: Figure 2(a)).

### Image analysis

DCE–MRI scans were processed to yield BBB leakage rate (*K_i_*) maps using a previously described method.^10^ In summary, the DCE–MRI images were motion corrected (FSL mcflirt^19^) and spatially coregistered (FSL flirt^19^) to the reference space (first post-contrast T1 map), then the vascular input function (VIF) was extracted from a manually selected volumetric region in the superior sagittal sinus (⩾188 mm^3^).^20^ Blood contrast agent concentrations were corrected for individual hematocrit levels, determined from venous blood samples obtained as part of the study assessment (*C*_plasma_ = *C*_blood_/(1 − hematocrit)). Tissue contrast-agent concentrations were calculated using a gadobutrol *r*_1_ relaxivity of 4.5 s^–1^mM^–1^.^21^ Here, the BBB transfer constant (*K*_trans_) is reported as *K_i_* after hematocrit correction, reflecting the unidirectional BBB transfer constant estimated using the graphical Patlak method.^15,22^ Because *K_i_* was estimated from two post-contrast Patlak points spaced >20 min apart, fit-quality exclusion was not applicable. Voxels with negative *K_i_* slope values were retained. Reported values are the voxel-wise mean per region.

The VAI scans were processed to yield maps of relative cerebral blood volume (CBV) and vessel size index (VSI), the weighted average of vessel radii within a voxel, using a previously published method (Supplemental Materials).^16^ The resulting parameter maps were coregistered to the reference space from DCE–MRI, allowing for the calculation of the vascular surface area (*S*) and the intrinsic BBB permeability (*P*) according to: *S* = 2CBV/VSI and *P* = *K_i_*/*S* (derivation: Figure 2(b)).

Brains were automatically segmented (software: SAMSEG)^23^ into the following regions of interest (ROI): deep gray matter (DGM), normal-appearing white matter (NAWM), and white matter hyperintensities (WMH), based on T1-weighted and FLAIR images. WMH masks were manually corrected if necessary and abnormalities like infarctions were manually segmented and excluded from the tissue ROIs. To evaluate distance-based (i.e. penumbra) effects on *P* and *S* adjacent to WMH, five concentric 2-mm-wide shells were defined surrounding WMH within the NAWM. To avoid partial-volume effects from the cerebrospinal fluid, a 3-mm periventricular rim (approximately equivalent to the in-plane resolution of the VAI) was removed from tissue masks. The cortex was excluded from analysis, as its relatively large pial vessels cause uncorrectable partial-volume effects at the VAI voxel size, disproportionately elevating VSI and reducing microvascular specificity.

### Statistical analysis

Data normality was evaluated with the Kolmogorov–Smirnov test. Between-group comparisons of demographic and clinical variables employed independent-samples *t*-tests (normally distributed data), Mann–Whitney *U* tests (non-normal), and χ^2^ tests (categorical data).

To evaluate the effects of tissue type, cSVD and age on each MRI measure (*K_i_*, *P*, and *S*), separate linear mixed-effects models were fitted. These included fixed effects for tissue type (reference: NAWM), disease status, age, and sex, interactions between cSVD and tissue type, and a random intercept per subject.

Finally, the spatial course of *K_i_*, *P*, and *S* surrounding WMH was determined using linear mixed-effect models with fixed effects for shell distance, disease status, age, and sex, and a random intercept per patient. A *p* < 0.05 was considered statistically significant.

## Results

Out of 99 enrolled participants, 71 were retained for analysis: 45 patients with cSVD and 26 elderly controls. A flowchart of participant inclusion is presented in Supplemental Figure S1. Participant characteristics are described in Table 1. WMH volumes were approximately six times larger in patients compared to controls.

**Table 1.** Participant characteristics (*N* = 71).

Characteristic	cSVD patients (*n* = 45)	Elderly controls (*n* = 26)	*p* Value
Female sex, *N* (%)	15 (33%)	6 (23%)	0.521
Age (years), mean (SD)	69.4 (9.5)	68.0 (6.5)	0.464
VCI, *N* (%)/lacunar stroke	29 (64%)/ 16 (36%)	0/0	**N/A**
MMSE, mean (SD)	27.0 (3.6)	29.0 (1.2)	**0.003**
BMI, mean (SD)	27.3 (4.1)	26.0 (3.2)	0.130
History of hypertension, *N* (%)	36 (80%)	15 (58%)	0.082
Systolic blood pressure (mmHg), mean (SD)	132.5 (18.2)	139.2 (20.8)	0.180
Diastolic blood pressure (mmHg), mean (SD)	86.4 (10.9)	87.0 (14.0)	0.862
eGFR (mL/min), mean (SD)	74.1 (13.6)	73.8 (12.0)	0.930
Diabetes, *N* (%)	6 (13%)	4 (15%)	1.000
Smoking history, *N* (%)	25 (56%)	16 (62%)	0.809
Hypercholesterolemia, *N* (%)	41 (91%)	14 (54%)	**0.001**
History of ischemic stroke, *N* (%)	27 (60%)	0 (0%)	**<0.001**
History of TIA *N* (%)	15 (33%)	3 (12%)	0.080
WMH volume, normalized to intracranial volume, mean (SD)	0.016 (0.014)	0.0025 (0.0028)	**<0.001**
WMH volume (cm^3^), mean (SD)	24.35 (20.47)	3.78 (4.14)	**<0.001**
Total Fazekas score (median, IQR)	4 (3–6)	1 (1–2)	**<0.001**

*N:* number, IQR: interquartile range, SD: standard deviation; BMI: body mass index; eGFR: estimated glomerular filtration rate; MMSE: mini-mental state examination; TIA: transient ischemic attack; VCI: vascular cognitive impairment.

Total Fazekas score: the sum of deep Fazekas score (0–3) and periventricular Fazekas score (0–3). Bold *p*-values indicate statistically significant differences between groups.

**Figure 3.**
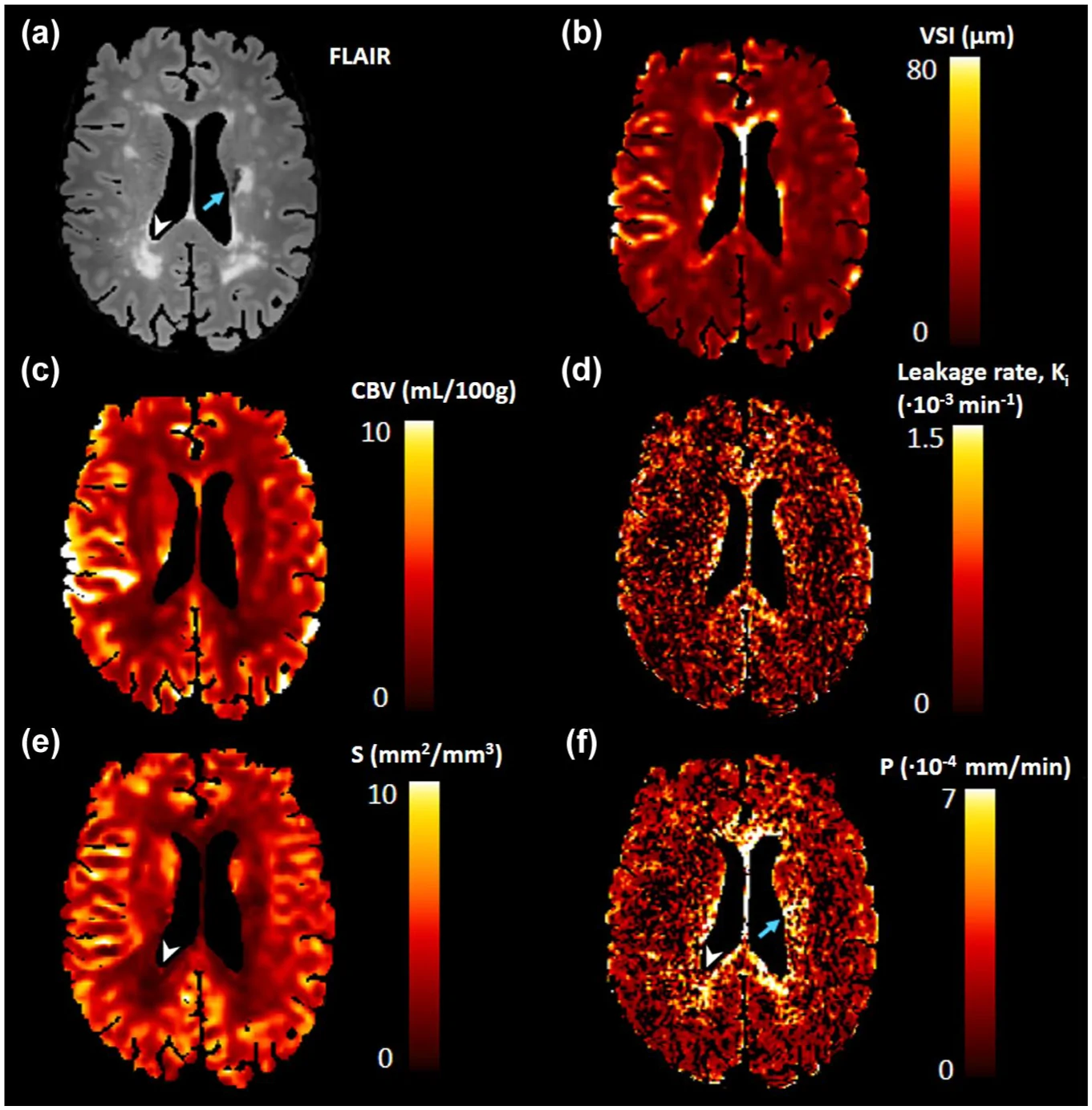
Brain maps for a cSVD patient (60 years old, male) showing: (a) white matter hyperintensities and a lacunar infarct on a T2 FLAIR image, (b) VSI, (c) CBV, and (d) blood–brain barrier leakage rate (*K_i_*), which can be split up in (e) vascular surface area (*S*) and (f) intrinsic vascular permeability (*P*). The white arrowhead shows decreased *S* in white matter hyperintensities, increasing conspicuousness on *P*, but not *K_i_* maps. The blue arrow points to an approximately 7-year-old infarct, whose surroundings show increased *P*, but not *K_i_*. CBV: cerebral blood volume; FLAIR: fluid-attenuated inversion recovery; VSI: vessel size index.

Descriptive statistics for *K_i_*, *P*, and *S* are provided in Supplemental Table S2. Figure 3 and Supplemental Figures S2 and S3 show examples of parameter maps. Regions with low *S* roughly colocalized with the larger WMH (a, e). *P* presented more conspicuously than *K_i_* near WMH and deep infarctions (d, f). DCE-derived blood plasma fraction (*v_p_*) showed low correspondence with VAI-derived VSI across tissue types (pooled *R*^2^ = 0.02, Supplemental Figure S4).

### Leakage, permeability, and surface area per tissue type

**Figure 4.**
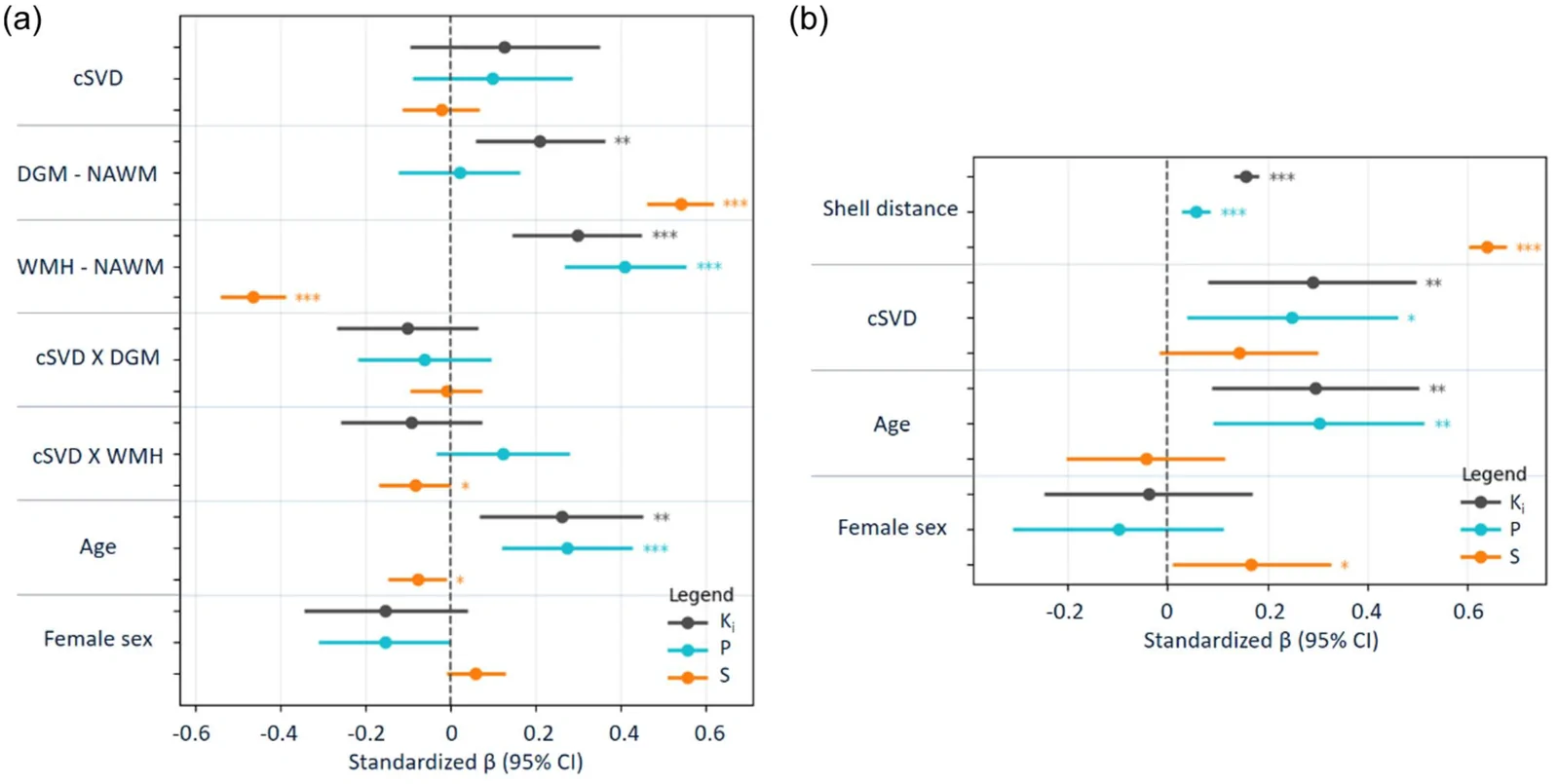
(a) Forest plot of leakage rate (*K_i_*), intrinsic permeability (*P*), and vascular surface area (*S*) in the DGM and WMHs in reference to NAWM of elderly controls and patients with cSVD evaluated using multivariable mixed-effects models for each measure and (b) forest plot of *K_i_*, *P*, and *S* across shells surrounding WMH, analyzed with multivariable mixed-effects models. cSVD: cerebral small vessel disease; DGM: deep gray matter; NAWM: normal appearing white matter; WMHs: white matter hyperintensities. *p < 0.05. **p < 0.01. ***p < 0.001.

Leakage rate *K_i_*, was higher in WMH (β = 0.297, *p* < 0.001) and DGM (β = 0.210, *p* = 0.007) than in NAWM. In contrast, permeability *P* did not differ between DGM and NAWM (β = 0.019, *p* = 0.796), whereas higher *P* was observed in WMH compared to NAWM (β = 0.410, *p* < 0.001). The surface area *S* was smaller in WMH (β = −0.467, *p* < 0.001) and larger in DGM (β = 0.540, *p* < 0.001) than NAWM (Figure 4(a)).

### Effect of cSVD and age

**Figure 5.**
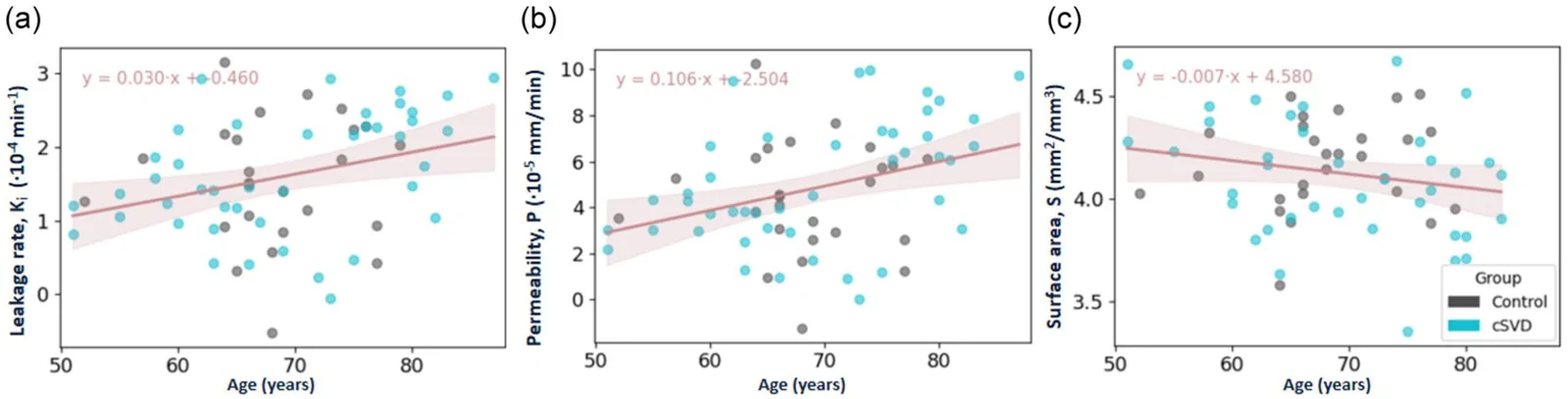
The relationship between age and (a) blood–brain barrier leakage rate, *K_i_*, (b) intrinsic blood–brain barrier permeability, *P*, and (c) vascular surface area, *S*, in the NAWM for patients and controls. The red line denotes the pooled regression line, with the shaded area being the 95% CI of that line. Influential outliers were removed (Cook’s *D* >4/*n*). NAWM: normal appearing white matter.

Comparing patients and controls, neither regionally averaged *K_i_* (all *p* ⩾ 0.224) nor *P* (all *p* ⩾ 0.124) differed significantly in any tissue type. However, *S*_WMH_ was smaller in patients with cSVD compared to controls (β = −0.085, *p* = 0.049), while no significant differences in *S*_DGM_ (β = −0.011, *p* = 0.797) and *S*_NAWM_ (β = −0.024, *p* = 0.604) were observed (Figure 4(a)). For all tissue types, *K_i_* as well as *P* increased with age (β = 0.260, *p* = 0.008 and β = 0.273, *p* < 0.001, respectively), while *S* decreased with age (β = −0.079, *p* = 0.025). Figure 5 shows how each parameter measured in NAWM varies with age.

### Distance effect from WMH boundary

**Figure 6.**
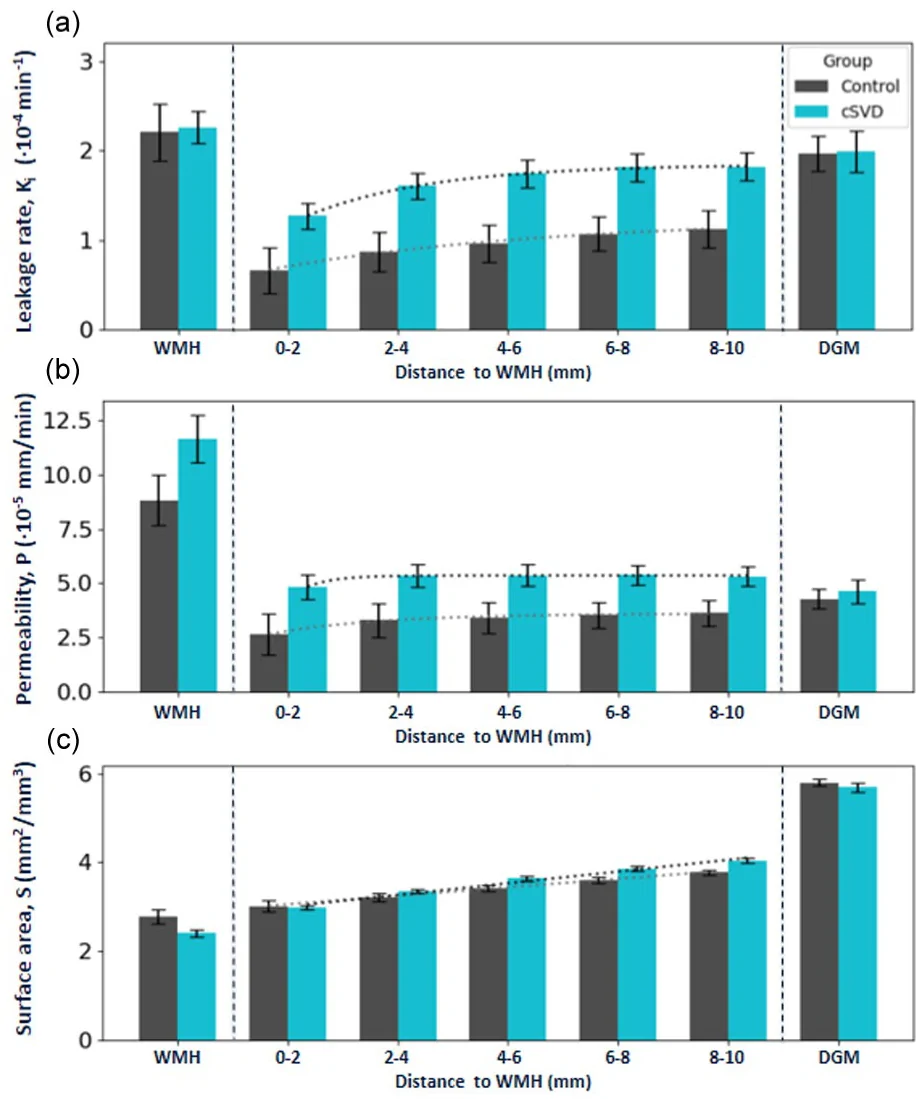
Spatial profile of: (a) blood–brain barrier leakage rate, *K_i_*, (b) intrinsic blood–brain barrier permeability, *P*, and (c) vascular surface area, *S*, in the WMHs, 2-mm-wide shells surrounding WMH, and in the DGM, including fit lines for patients and controls (mean, standard error). DGM: deep gray matter; WMHs: white matter hyperintensities.

In perilesional NAWM, *K_i_*, *P*, and *S* each increased with distance from the WMH (all *p* < 0.001; Figure 6). In the perilesional shells, both *K_i_* and *P* were significantly higher in patients than controls (β = 0.289, *p* = 0.007 and β = 0.249, *p* = 0.021, respectively). Perilesional *S* was similar between groups (β = 0.142, *p* = 0.078; Figure 4(b)). Supplemental Figure S5 shows patients included with VCI and those with a lacunar infarction are comparable across measures, as all standard errors overlap, with the exception of lower *S* in DGM for VCI.

## Discussion

In this study, we were able to disentangle the MRI-derived blood–brain barrier leakage rate of cerebral blood vessels into their intrinsic vascular permeability and surface area. In patients with cSVD and elderly controls, disentangling the concomitant opposing effects of reducing surface area and increasing permeability (e.g. with age or disease) provides a biologically more clear and specific view of the underlying BBB pathophysiology.

Across brain tissue types, MRI-derived surface area *S* followed a clear spatial hierarchy, decreasing from DGM to NAWM, and WMH, in accordance with histological studies of microvessel density.^24,25^ Similar intrinsic permeability *P* in DGM and NAWM confirmed that the stronger leakage (*K_i_*) in (D)GM than NAWM is due to differences in *S*, as commonly hypothesized, but up till now never explicitly measured.^5,6^ Higher BBB permeability and smaller vascular surface area, as seen for instance in WMH, counteract each other as opposing effects when captured by *K_i_*. Consequently, areas with increased BBB disruption are much better visualized by mapping *P* than *K_i_* (Figure 3 and Supplemental Figure S2). Considering the apparent noise on *K_i_* maps, Supplemental Figure S2 shows *P* maps also visualize hotspots more conspicuously than *K_i_* maps down-sampled from 1 to the 5-mm VAI slice thickness. Compensatory effects of vasodilation and vascular remodeling would explain why no difference in *S* between patients and controls was found in NAWM and DGM. By contrast, in WMH this compensation may have become insufficient. Only in the perilesional NAWM were both *K_i_* and *P* higher in patients compared to controls. As expected, both *K_i_* and *P* increase, while *S* decreases with age (Figure 5).^26,27^ Per decade, *S* decreases 3.1% (0.13 mm^2^/mm^3^) and *P* increases 37.2% (1.78 × 10^−4^ mm/min) of the mean values over all participants in the NAWM, respectively.

*K_i_* increased perilesionally with distance from WMH, which was largely driven by increases in *S*, as *P* increased less with distance (β_*P*_ = 0.057 vs β_*ki*_ = 0.158) and plateaued earlier for both patients and controls. Recent studies have shown increased vasodilation and capillary to arteriole remodeling in response to local WMH-related hypoperfusion in neurodegenerative and cerebrovascular disease.^16,28^ Our observation of larger *S* in the WMH penumbra therefore suggests (preserved) mechanisms to compensate perfusion losses just outside of WMH, where compensation fails after transition to WMH. This failure appears to be more severe in patients with cSVD compared to elderly controls.

Currently, only secondary prevention strategies exist for cSVD and disease modifying treatments are lacking. Our method should allow for recruitment of patients for future clinical trials^29^ based on their microvascular status, depending on the targeted modifiable factor (*P* or *S*) and who should derive the most benefit. *P* also has the potential to be a biologically much more specific measure to assess treatment efficacy in early stages compared to *K_i_*, as vascular surface area may be changing over time and at different rates per individual. This same specificity to intrinsic BBB permeability can also be beneficial for drug-delivery research.^30^

Some potential limitations should be considered. Our approach requires an additional VAI acquisition with gadolinium administration beyond standard *K_i_* mapping, trading 3 min of scan time for greater specificity to intrinsic permeability (*P*). The VAI voxel size of 3 × 3 × 5 mm is comparable to the cortical thickness, and thus contains a large contribution from pial vessels, leading us to exclude the cortical gray matter. Cortical GM may show microvascular and BBB permeability changes distinct from the DGM in cSVD and aging due to differences in vascular architecture. Future VAI sequences require higher spatial resolution to allow distinction between pial vessels and cortical GM. The spatial resolution also impeded accurate determination of the arterial input function, which had to be scaled by reference rCBV values for the NAWM. As DCE–MRI derived blood plasma fraction (*v_p_*) did not differ in the NAWM of patients and controls in this cohort, we expect group-wise bias to be limited.^10,31^ Additionally, the patient-control comparisons should be interpreted cautiously because of the modest sample size, multiple statistical comparisons, and potential loss of sensitivity from VAI partial-volume effects propagating into *P*. Marginal findings, such as lower *S* in WMH of patients than controls, require confirmation in larger studies, while non-significant group differences should not be taken as evidence that subtle effects are absent. Finally, histological reference values for *S* in cortical GM (which has comparable vessel density to DGM^25^) range from 11.7 mm^2^/mm^3^ obtained by 3D reconstruction of (India-ink-perfused) vessels imaged with confocal microscopy in a single human brain,^32^ to approximately 16.4 mm^2^/mm^3^ in postmortem prefrontal cortical GM from 36 individuals, estimated stereologically from (Glut-1) stained vessel walls.^33^ The latter study additionally reported an *S* of approximately 11.3 mm^2^/mm^3^ in WM.^33^ The considerable difference between the two histological estimates, obtained using different techniques, illustrates the lack of a definitive gold-standard reference value. Our method measured *S* in DGM at 5.7 mm^2^/mm^3^ and in NAWM at 4.1 mm^2^/mm^3^, reflecting an underestimation, that is, likely caused by vessels wider than capillaries (e.g. arterioles and venules) contributing significantly to the weighted mean radius (VSI), while contributing much less to *S* than capillaries. The observed surface-area ratio of 1.4 between DGM and NAWM was relatively close to the study evaluating *S* in both GM and WM, which reported a ratio of 1.6 for vascular surface-area density in prefrontal GM and WM.^33^ However, future paired MRI-histology comparison with samples containing both capillaries and larger vessels is needed to validate any tissue-specific bias. Adding a radius distribution to the VSI, with a technique like vessel distribution imaging (VDI) or a correction for lower sensitivity to the thinnest vessels, could provide an unweighted mean and be a future refinement step for estimating *S*.^34^

In conclusion, splitting the rate of contrast agent leakage, *K_i_*, into *P* and *S*, provides a more specific view of the underlying biology of the vasculature and its pathophysiological changes, as opposite effects of rarefaction (decreased *S*) and BBB breakdown (increased *P*) on *K_i_* counteract one another (Figure 1). Biologically, reductions in *S* represent less opportunity to exchange nutrients and oxygen between blood and parenchyma, whereas increased *P* reflects a malfunction of the BBB’s protection mechanism. On the other hand, the leakage rate itself may be a biomarker of the potential influx of harmful substances from the blood into the brain parenchyma. This study demonstrates a method to disentangle these biologically distinct alterations underlying contrast agent leakage and enhancement in brain disease and aging.

## Supplemental Material

sj-docx-1-jcb-10.1177_0271678X261485415 – Supplemental material for Disentangling MRI-derived blood–brain barrier leakage into vascular permeability and surface area Supplemental material, sj-docx-1-jcb-10.1177_0271678X261485415 for Disentangling MRI-derived blood–brain barrier leakage into vascular permeability and surface area by Damon Verstappen, Joost JA de Jong, Paulien HM Voorter, Maud van Dinther, Robert J van Oostenbrugge, Julie Staals, Jacobus FA Jansen and Walter H Backes in Journal of Cerebral Blood Flow & Metabolism
