# Supplementary material for: Disentangling MRI-derived blood–brain barrier leakage into vascular permeability and surface area
Source: J Cereb Blood Flow Metab. 2026 Aug 26:0271678X261485415. Online ahead of print. doi: 10.1177/0271678X261485415 (PMC13586075; doi:10.1177/0271678X261485415)
Supplement: Supplementary file 1 [file sj-docx-1-jcb-10.1177_0271678X261485415.docx]

**Supplementary materials**

**Supplementary methods**

Calculation of VSI and rCBV

First, the dynamic GE and SE images were motion corrected (FSL mcflirt^1^) and corrected for geometric distortions (FSL topup^2^). Then the dynamic change in R_2_* and R_2_ per ROI was calculated by dividing their signals by the average pre-contrast signals (S_0_): ∆R_2_*(t)=-ln(S_GE_(t)/S_GE,0_)/TE_GE_ and ∆R_2_(t)=-ln(S_SE_(t)/S_SE,0_)/TE_SE_. The VSI is calculated as follows^3^: VSI=0.867∙(D∙rCBV)^1/2^∙Q^-2/3^. In this calculation, D is the parenchymal diffusion coefficient, derived from a mono-exponential function fit of the diffusion signal decay for b ≥ 200 s/mm^2^.^4^ The rCBV denotes the relative cerebral blood volume calculated from the GE images according to the method of Kellner, et al.^5^ Q denotes the vessel density index, defined as: Q=∆R_2_/(∆R_2_*)^2/3^, using the 4 time points before, and 6 time points after the first bolus peak.^3^

**Table S1:** MRI acquisition protocol

|  | Double inversion-time GE | First-pass DCE | Vessel Architecture Imaging | Diffusion-weighted imaging |
| --- | --- | --- | --- | --- |
| Sequence type | Inversion Recovery GE | 3D Fast-Field Echo | rapid transverse single-shot combined GE SE EPI | single shot SE EPI inversion-recovery |
| Repetition time (ms) | 8.3 | 5.3 | 1634 | 6800 |
| Echo time (ms) | 3.8 | 1.6 | GE=25, SE = 78 | 86 |
| Inversion time 1 (ms) | 650 | N/A | N/A | 2230 |
| Inversion time 2 (ms) | 2100 | N/A | N/A | N/A |
| Flip angle | 6° | 30° | 90° | 90° |
| b-values (s/mm^2^) | N/A | N/A | N/A | 0, 200, 300, 400, 500, 600, 800, 1000 |
| Matrix size | 240 x 240 | 128 x 128 | 76 x 61 | 112 x 112 |
| Number of slices | 180 | 21 | 16 | 56 |
| Voxel size (mm) | 1 x 1 x 1 | 2 x 2 x 6 | 3 x 3.3 x 5 | 2.4 x 2.4 x 2.4 |
| Slice gap (mm) | N/A | N/A | 2 | 0.48 |
| k-space ordering | Center | Center | EPI | EPI |
| Frequency encoding direction | Superior-Inferior | Right-Left | Right-Left | Right-Left |
| Dynamic scans | N/A | 70 | 100 | N/A |
| Dynamic scan interval (s) | N/A | 1.46 | 1.6 | N/A |
| Contrast agent dose (mL/kg body weight) | N/A | 0.1 | 0.1 | N/A |
| Contrast agent injection rate (mL/s) | N/A | 3 | 3 | N/A |
| Total acquisition time (minutes: seconds) | 4:36 | 2:09 | 2:50 | 5:28 |

GE = Gradient echo, SE = spin echo, EPI = Echo planar imaging

**Figure S1:** Flowchart describing participant inclusion. N = number, VAI = vessel architecture imaging, DSC = dynamic susceptibility contrast, cSVD = cerebral small vessel disease

**Table S2:** Descriptive values for blood-brain barrier (BBB) leakage rate (K_i_), intrinsic BBB permeability (P), and vascular surface area (S), split for patients with cerebral small vessel disease (cSVD) and controls. Notation: Mean (standard deviation)

| Measure | cSVD | Control |
| --- | --- | --- |
| **K_i_ (∙10^-4^ min^-1^)** | | |
| DGM | 1.99 (1.55) | 1.97 (1.02) |
| NAWM | 1.75 (0.95) | 1.40 (1.11) |
| WMH | 2.26 (1.23) | 2.21 (1.61) |
| **P (∙10^-5^ mm/min)** | | |
| DGM | 4.61 (3.65) | 4.27 (2.29) |
| NAWM | 5.22 (2.91) | 4.05 (3.04) |
| WMH | 11.66 (7.33) | 8.82 (5.98) |
| **S (mm^2^/mm^3^)** | | |
| DGM | 5.68 (0.75) | 5.79 (0.40) |
| NAWM | 4.10 (0.37) | 4.17 (0.23) |
| WMH | 2.41 (0.53) | 2.77 (0.80) |

**Figure S2**: Brain maps for a cSVD patient (82 years old, female) showing: (a) white matter hyperintensities on a T2 fluid-attenuated inversion recovery (FLAIR) image, (b) vessel size index (VSI), (c) cerebral blood volume (CBV), (d) blood-brain barrier leakage rate (K_i_), which can be split up in (e) vascular surface area (S) and (f) intrinsic vascular permeability (P). (g) Shows the information included in the 5 mm thick VAI slab, by averaging the FLAIR signal across that slab. (h) Shows K_i_ averaged across the VAI slab.

**Figure S3**: Brain maps for a cognitively normal control (68 years old, male) showing: (a) white matter hyperintensities on a T2 fluid-attenuated inversion recovery (FLAIR) image, (b) vessel size index (VSI), (c) cerebral blood volume (CBV), (d) blood-brain barrier leakage rate (K_i_), which can be split up in (e) vascular surface area (S) and (f) intrinsic vascular permeability (P)

**Figure S4.** Relationship between DCE-derived blood plasma fraction (v_p_) and VAI-derived vessel size index (VSI) per tissue type.

**Figure S5:** Spatial profile of: (a) blood-brain barrier leakage rate, K_i_, (b) intrinsic blood-brain barrier permeability, P, (c) vascular surface area, S, in the white matter hyperintensities (WMH), 2-milimetre-wide shells surrounding WMH, and in the deep gray matter (DGM), split by inclusion criterion, either due to vascular cognitive impairment (VCI), having a lacunar infarction or being a control (mean, standard error).

**References**

1.	Jenkinson M, Bannister P, Brady M, Smith S. Improved Optimization for the Robust and Accurate Linear Registration and Motion Correction of Brain Images. *NeuroImage*. 2002;17:825-841. doi: <https://doi.org/10.1006/nimg.2002.1132>

2.	Andersson JLR, Skare S, Ashburner J. How to correct susceptibility distortions in spin-echo echo-planar images: application to diffusion tensor imaging. *NeuroImage*. 2003;20:870-888. doi: <https://doi.org/10.1016/S1053-8119(03)00336-7>

3.	Troprès I, Pannetier N, Grand S, Lemasson B, Moisan A, Péoc'h M, Rémy C, Barbier EL. Imaging the microvessel caliber and density: Principles and applications of microvascular MRI. *Magn Reson Med*. 2015;73:325-341. doi: 10.1002/mrm.25396

4.	Paschoal AM, Leoni RF, Dos Santos AC, Paiva FF. Intravoxel incoherent motion MRI in neurological and cerebrovascular diseases. *Neuroimage Clin*. 2018;20:705-714. doi: 10.1016/j.nicl.2018.08.030

5.	Kellner E, Breyer T, Gall P, Müller K, Trippel M, Staszewski O, Stein F, Saborowski O, Dyakova O, Urbach H, et al. MR evaluation of vessel size imaging of human gliomas: Validation by histopathology. *Journal of Magnetic Resonance Imaging*. 2015;42:1117-1125. doi: <https://doi.org/10.1002/jmri.24864>
